# Editorial: Exercise and cancer: From clinical association to mechanistic insights

**DOI:** 10.3389/fmolb.2022.1007228

**Published:** 2022-09-06

**Authors:** Yao Lin, Hang Fai Kwok

**Affiliations:** ^1^ Central Laboratory at the Second Affiliated Hospital of Fujian Traditional Chinese Medical University, Fujian-Macao Science and Technology Cooperation Base of Traditional Chinese Medicine-Oriented, Collaborative Innovation Center for Rehabilitation Technology, Fujian University of Traditional Chinese Medicine, Fuzhou, China; ^2^ Cancer Centre, Faculty of Health Sciences, University of Macau, Taipa, Macao SAR, China; ^3^ Department of Biomedical Sciences, Faculty of Health Sciences, University of Macau, Taipa, Macao SAR, China; ^4^ MoE Frontiers Science Center for Precision Oncology, University of Macau, Avenida de Universidade, Taipa, Macao SAR, China

**Keywords:** physical exercise (EX), cancer, chronic disease, treatment, prevention

Cancer is one of the leading causes of mortality worldwide. The association between physical exercise and many aspects of cancer, such as incidence and prognosis, has been well documented. In addition, many risk factors of cancer including obesity, aging, and inflammation are affected by physical exercise. Although it is widely recognized that physical exercise has a positive effect on cancer in terms of morbidity, prognosis, rehabilitation, and even therapy, there are issues in this field awaiting a deeper understanding. In this research topic, biological and biomedical scientists summarized or investigated the latest progress in multiple areas of this field.

Four studies investigated the effect of exercise in different cancer models. Suzuki et al. carried out an interesting research on the combined effects of exercise training and nutritional supplementation in cancer patients in the context of coronavirus (COVID-19). They proposed that combining dietary supplements and exercise training in cancer patients can boost immune responses against COVID-19 and probably improve vaccine responses. Li et al. evaluated the impact of swimming on murine colon cancer cell line CT-26 xenograft model. Swimming significantly attenuates tumor growth and muscle wasting, and suppresses inflammatory and apoptosis pathways. Kim et al. also used CT-26 cells to study the effect of high-intensity aerobic exercise on cancer (https://doi.org/10.3389/fmolb.2022.818470). Instead of using xenograft, they injected CT-26 cells via the tail vein to establish a cancer mouse model. They discovered that exercise improved positive results in comprehensive parameters such as food intake, weight gain and survival rate. Jin et al. carried out a meta-analysis of animal experiments to study the effects of exercise on breast cancer (https://doi.org/10.3389/fmolb.2022.843810). Based on their analyses, exercise could reduce tumor weight, the number of tumors per animal, and the tumor incidence in breast cancer models of mice and rats. However, the standards of conducting and reporting animal works need to be improved.

In addition, another five studies investigated exercise and cancer from other angles. Zhu et al. studied the physical activity and cancer status among middle-aged and older Chinese. They reported that individuals who spent more than half an hour performing moderate or vigorous intensity activity every day were significantly less likely to report a cancer diagnosis than inactive individuals. Chen et al. constructed a modified model to predict malignancy in thyroid nodules with small size using ultrasound characters (https://doi.org/10.3389/fmolb.2021.752417), which may provide useful tools to evaluate the effect of exercise on early tumor development. Wu et al. investigated the association of the methylation and expression of the exercise-related toll like receptor-1 (TLR-1) gene with the prognosis and outcome of low-grade glioma (LGG). They found that TLR-1 can be a potential prognostic marker and may be involved in immune cell infiltration and immunotherapy in LGG. Ochi et al. analyzed the blood of patients from a 12-weeks trial to evaluate the effect of exercise on cancer-related fatigue (CRF). They suggested that blood polyunsaturated fatty acid (PUFA) balance may be associated with the effect of exercise on CRF. Jin et al. carried out a bibliometrics study on the molecular mechanisms of exercise on cancer. The authors discovered that altered metabolism, oxidative stress and apoptosis were current research hot spots in this field, and emerging research foci were generally around inflammation, epithelial mesenchymal transition (EMT) and adipokines.

Although covering a relatively broad range in the cancer field, it is a pity this research topic did not have any work on the bioactive material mediating the effect of exercise on cancer, which is an emerging promising direction in this field. For example, Bar-sagi et al. discovered that exercise-induced activation of the IL-15/IL-15Rα pathway promotes anti-tumor immunity in pancreatic cancer (Kurz et al., 2022). Likewise, Saxton et al. found that acute aerobic exercise-conditioned serum reduces colon cancer cell proliferation *in vitro* through interleukin-6 (Orange et al., 2022).

There is no doubt more such intermediate bioactive molecules such as proteins, miRNAs, metabolites and so on will be identified in the future. They are probably the key to unravel the complex functions of exercise on cancer. The deepening of our understanding of the mechanisms underlying the effect of exercise on cancer may 1 day help us harness the benefits of exercise without actually exercising.
